# Potential Actions of Baicalein for Preventing Vascular Calcification of Smooth Muscle Cells In Vitro and In Vivo

**DOI:** 10.3390/ijms23105673

**Published:** 2022-05-18

**Authors:** Erna Sulistyowati, Jong-Hau Hsu, Szu-Jung Lee, Shang-En Huang, Widya Yanti Sihotang, Bin-Nan Wu, Zen-Kong Dai, Ming-Chung Lin, Jwu-Lai Yeh

**Affiliations:** 1 Graduate Institute of Medicine, College of Medicine, Kaohsiung Medical University, Kaohsiung 807, Taiwan; dr_erna@unisma.ac.id (E.S.); jhh936@yahoo.com.tw (J.-H.H.); roung510@yahoo.com.tw (S.-J.L.); eva_1433@yahoo.com.tw (S.-E.H.); widyayantisihotang@unprimdn.ac.id (W.Y.S.); binnan@kmu.edu.tw (B.-N.W.); zenkong@kmu.edu.tw (Z.-K.D.); 2 Faculty of Medicine, University of Islam Malang, Malang 65145, Indonesia; 3 Department of Pediatrics, College of Medicine, Kaohsiung Medical University, Kaohsiung 807, Taiwan; 4 Department of Pediatrics, Kaohsiung Medical University Hospital, Kaohsiung 807, Taiwan; 5 Faculty of Public Health, Prima University of Indonesia, Medan 20118, Indonesia; 6 Department of Pharmacology, College of Medicine, Kaohsiung Medical University, Kaohsiung 807, Taiwan; 7 Department of Medical Research, Kaohsiung Medical University Hospital, Kaohsiung 807, Taiwan; 8 Department of Anesthesiology, Chi Mei Medical Center, Tainan 710, Taiwan; 9 Department of Marine Biotechnology and Resources, National Sun Yat-sen University, Kaohsiung 804, Taiwan

**Keywords:** osteogenic differentiation, phenotypic switch, apoptosis, oxidative stress

## Abstract

Vascular calcification (VC) is associated with cardiovascular disease. Baicalein, a natural flavonoid extract of *Scutellaria baicalensis* rhizome has several biological properties which may inhibit VC. We investigated whether baicalein suppresses Runt-related transcription factor 2 (Runx2) and bone morphogenetic protein 2 (BMP-2) and upregulates smooth muscle 22-alpha (SM22-α) and alpha-smooth muscle actin (α-SMA). In an in vitro experiment, primary rat aortic vascular smooth muscle cells (VSMCs) were pretreated with 0.1, 1, and 5 μM baicalein, followed by β-glycerophosphate (β-GP) to induce calcification. In an in vivo experiment, VC was generated by vitamin D3 plus nicotine (VDN) administration to male Sprague Dawley (SD) rats randomly assigned into a control group, a VC group, a VC group pretreated with baicalein, and a baicalein alone group. Each group comprised 10 rats. Left ventricular (LV) morphology, function and performance were assessed by echocardiography. Calcium content was measured by Alizarin red S staining and alkaline phosphatase (ALP) activity assays. Apoptotic VSMCs were detected by flow cytometry. Protein levels and superoxide changes were evaluated using Western blotting and immunofluorescence assays respectively. Plasma malondialdehyde (MDA) was assayed. Baicalein pretreatment significantly reduced calcium content in calcified VSMCs (*p* < 0.001) as well as in VC rat aortic smooth muscle (*p* < 0.001). Additionally, ALP activity was decreased in calcified VSMCs and VC rat aortic smooth muscle (*p* < 0.001). Apoptosis was significantly attenuated by 1 μM baicalein pretreatment in calcified VSMCs. Runx2 and BMP-2 expressions were downregulated by the baicalein in calcified VSMCs. Baicalein pretreatment increased typical VSMCs markers SM22-α and α-SMA in calcified VSMCs. Baicalein pretreatment was associated with adverse changes in LV morphometry. Markers of oxidative stress declined, and endogenous antioxidants increased in VC rats pretreated with baicalein. Baicalein mitigates VC through the inhibition of Runx2/BMP-2 signaling pathways, enhancement of vascular contractile phenotype and oxidative stress reduction. However, our study is of basic experimental design; more advanced investigations to identify other molecular regulators of VC and their mechanisms of action is required.

## 1. Introduction

Vascular calcification is common in patients with hypertension, atherosclerosis, and diabetes [1]. Although the precise relationship between VC and subsequent vascular events remains unclear, there is a clear correlation between atherosclerotic calcification and plaque vulnerability [2]. Emerging evidence demonstrates that vascular calcification is an actively regulated process and shares many features with bone development and metabolism. Several major pathways of vascular calcification include (1) failed anti-calcific processes [3], (2) induction of osteo-chondrogenesis [4], (3) cell death [5], (4) abnormal calcium and phosphate (Ca/Pi) homeostasis [6], (5) circulating calciprotein particles [7], and (6) matrix degradation/modification [3]. Apoptosis of VSMC plays a key role in vascular calcification, causing vessel wall stiffness and decreased contractile function. Several studies have confirmed that inhibition of apoptosis can significantly decrease both calcifying vesicle release and calcification [1]. In recent years, there has been growing research interest in bone morphogenetic protein 2 (BMP-2), an essential osteogenic protein required for osteoblast differentiation and bone formation, which has been implicated in vascular calcification. BMP-2 stimulates phosphate-induced smooth muscle cell calcification through the mechanism of Pit-1-related phosphate transporter activation. Furthermore, BMP-2 has an important role in the stimulation of Runx2 and inhibition of SM22-α expressions. This means that BMP-2 promotes osteogenic phenotype transition in VSMCs. In consequence, BMP-2 drives vascular calcification via increased phosphate uptake and initiation of osteogenic phenotype modulation in VSMCs [8].

The key factors that drive phenotypic change of VSMCs during vascular calcification are premature vascular aging and VSMC senescence [9]. The main stimulation of cellular senescence is oxidative stress, a state when the production of reactive oxygen species is increased beyond the capacity of antioxidant mechanisms. Oxidative stress is believed to lead to the impairment of vital cellular substructures including DNA, protein, and membrane/cytoplasmic lipids. The calcified VSMCs exhibit high levels of reactive oxygen species (ROS) production by cellular enzymes including xanthine oxidase, nicotinamide adenine dinucleotide phosphate oxidase (NADPH oxidase), nitric oxide synthase (NOX), and the cytochrome P450 systems. These ROS cause downstream events such as telomere shortening, mitochondrial deoxyribonucleic acid (DNA) damage, and direct mutagenic influences on gene fragments involved in contractile functions, resulting in aging of VSMCs and partial loss of smooth muscle cell phenotype. Oxidative stress can lead to the oxidative damage to VSMC and contributes phenotypic switching of VSMCs into osteoblast-like cells with down-regulated contractile marker expression (e.g., SM22α and calponin) and upregulated osteogenic marker expression (e.g., Runx2 and collagen I) [10,11]. Consequently, these features cause the activation of osteogenic transcription factor [12,13].

In the hope of preventing and treating vascular disease, modern nutritional science recommends intake of dietary phytochemicals which appear to benefit cardiovascular health. Flavonoids are found throughout the plant kingdom and numerous studies have evaluated their oral bioavailability, metabolism, and biological activity. One of the flavonoid subclasses is the flavones, found in significant concentrations in traditional diets of China and Korea [14]. Baicalein is one such flavone believed to have beneficial effects on cardiovascular health. 

Baicalein, (5,6,7-trihydroxy-2-phenyl-4H-1-benzopyran-4-one), extracted from the rhizome of the Chinese herb *Scutellaria baicalensis* Georgi. (Lamiaceae) has been shown to possess anti-inflammatory [15], antioxidant [16], anticancer [17], and antidiabetic properties [18]. Furthermore, baicalein may help to prevent cardiovascular related diseases such as cardiomyocyte injury (hypertension [19], oxidative stress-induced [20], and hyperglycemia-induced VSMC proliferation and migration [21]. Hence, it is speculated that baicalein may inhibit biochemical processes leading to vascular calcification by suppression of bone morphogenetic genes such as BMP-2 and osteogenic transcription factor gene such as Runx2. However, it has not yet been established whether baicalein can inhibit these genes in association with VC. We hypothesize that baicalein inhibits VC by inhibition of VSMC apoptosis, suppression the expression of Runx2 and BMP-2, and enhancement of vascular contractile-associated phenotype. In addition, we evaluated whether baicalein prevents cardiac remodeling and vascular oxidative stress.

In the current in vitro and in vivo studies, we used baicalein to inhibit vascular calcification processes by inhibiting the expression of Runx2 and BMP-2. Furthermore, VSMC phenotypic changes were assessed by measurement of the expression of SM22-α, an important VSMC contractile phenotypic marker. Heart weight, body weight, heart to body weight index, left ventricular internal diameter end diastole (LVIDd), left ventricular internal diameter end systole (LVIDs), and interventricular septal end diastole (IVSD) were measured, as were oxidative stress markers in the rat vascular wall. 

## 2. Results

### 2.1. Effect of Baicalein in the Inhibition of β-GP-Induced VSMC Calcification

**Figure 1:**
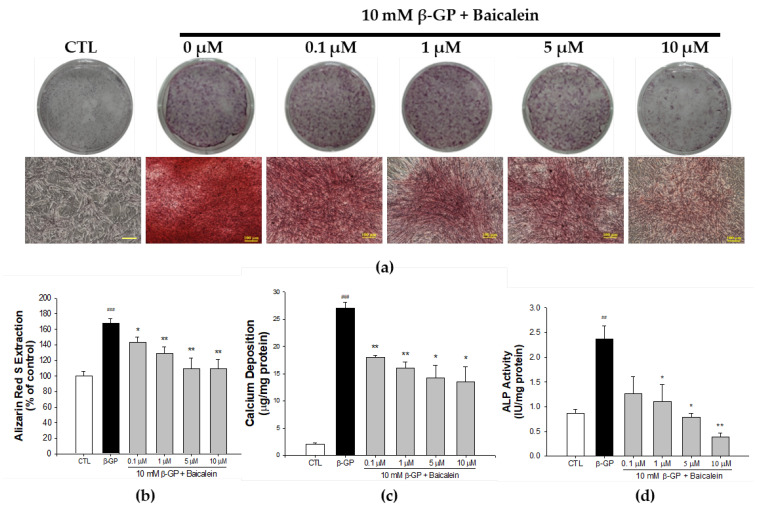
Baicalein decreases mineralization, calcium deposition and downgrades ALP activity in primary rat VSMCs. The cells were cultured with indicated concentrations of baicalein in the presence of β-glycerophosphate (β-GP)-induced calcification medium for 10 days. (**a**) Calcium nodules were observed under light microscope with Alizarin red S staining. (**b**) Red nodules are calcium deposition in cells culture and represent the amount of calcium. (**c**) In vitro calcium deposition level and (**d**) ALP activity in β-GP-induced primary rat VSMCs. Scale bar indicates 100 μm. All values are presented as mean ± SEM from four independent experiments. ^##^ *p* < 0.01; ^###^ *p* < 0.001 vs. control group (untreated VSMCs); * *p* < 0.05; ** *p* < 0.01 vs. the β-GP group.

To investigate the effect of baicalein on β-GP-induced calcification, VSMCs were incubated with calcification medium (10 mM β-GP) in the presence or absence of baicalein (0.1, 1, 5, and 10 μM). After 8 days of treatment, the VSMC calcification cell model was successfully established, demonstrating that baicalein significantly blocked calcium deposition, as shown by Alizarin red S staining (Figure 1).

### 2.2. Effect of Baicalein in Blocking Calcium Deposition and ALP Activity

**Figure 2:**
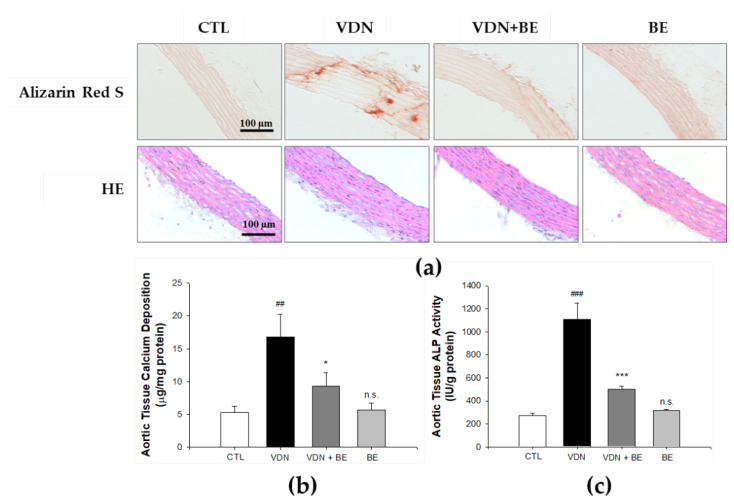
Baicalein reduces calcium deposition and downgrades ALP activity in thoracic aorta of SD rats. (**a**) The aortic calcium content examined by Alizarin red S staining. (**b**) Quantification the calcium level and (**c**) ALP activity in the thoracic aorta homogenates of SD rats. Scale bar indicates 100 μm. All values are presented as mean ± SEM from four independent experiments. ^##^ *p* < 0.01; ^###^ *p* < 0.001 vs. control group; * *p* < 0.05; *** *p* < 0.001 vs. the VDN group; n.s. mean not significant vs. control group.

An inhibitory effect of baicalein on calcium deposition was also found by Quantichrom^TM^ Calcium Assay kit method. As shown in Figure 1b, the level of mineralization in the β-GP group was 26.98 ± 1.09 μg/mg protein, compared with the control 2.07 ± 0.17 μg/mg protein (*p* < 0.001). Pretreatment with 0.1 μM baicalein reduced the calcium level to 18.01 ± 0.39 μg/mg protein, 1 μM baicalein reduced it to 15.99 ± 1.18 μg/mg protein, 5 μM baicalein reduced it to 14.22 ± 2.27 μg/mg protein, and 10 μM baicalein reduced it to 13.49 ± 2.8 μg/mg protein. As shown in Figure 1c, baicalein pretreatment (1, 5, and 10 μM) significantly reduced the level of ALP activity in β-GP-induced VSMCs calcification in a dose-dependent manner. Furthermore, to establish a VC rat model, we gave intramuscular injection of 3 × 10^5^ IU/kg body weight (BW), vitamin D3 plus oral 25 mg/kg nicotine in 5 mL corn oil in SD rats (VDN model). As presented in Figure 2b. baicalein treatment reduced the calcium content in aorta tissue of rats subjected to VDN-induced VC. The calcium level of the VDN group was 15.06 ± 2.43 μg/mg protein, compared with the control calcium level of 5.02 ± 0.07 μg/mg protein (*p* < 0.001). Pretreatment with 25 mg/kgBW baicalein reduced calcium level to 9.23 ± 0.92 μg/mg protein. Moreover, as shown in Figure 2c, the ALP activity in VDN-induced rat VC was 1106 ± 142 IU/g protein. Pretreatment with baicalein significantly decreased ALP activity level to 503 ± 23 IU/g protein (*p* < 0.001). These findings show that baicalein inhibits calcium deposition and ALP activity level both in vitro and in vivo.

### 2.3. Effect of Baicalein in Protecting VSMCs against β-GP-Induced Apoptosis 

**Figure 3:**
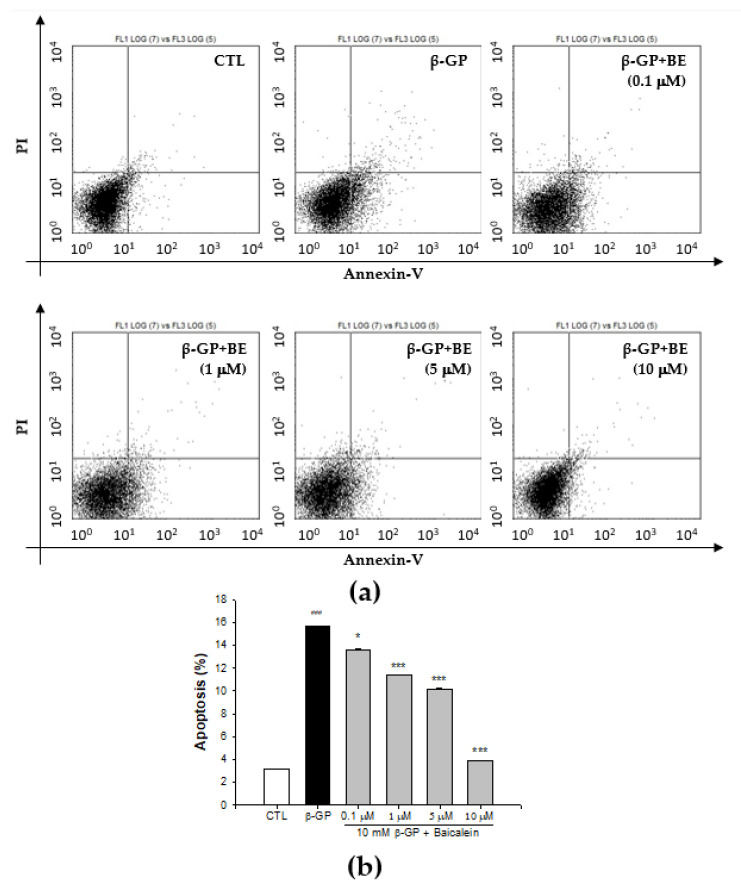
Baicalein inhibits apoptosis in primary rat VSMCs. The cells were pretreated with indicated concentrations of baicalein for 1 h before addition of 10 mM β-GP-induced calcification for 48 h. (**a**) The cells apoptosis determined by flow cytometry with Annexin V-PI staining. (**b**) The percentage of cells apoptosis. Data were representative of three independent experiments with 4 replicas per condition in each experiment. All values are presented as mean ± SEM from four independent experiments. ^###^ *p* < 0.001 vs. control group (untreated VSMCs); * *p* < 0.05, *** *p* < 0.001 vs. the β-GP group.

To detect apoptosis, we initially stained the VSMCs with Annexin V and propidium iodide solution followed by flow cytometry analysis. As shown in Figure 3, in the comparison with control group (3.13 ± 0.06%), the calcification group had a markedly increased number of apoptotic cells (15.63 ± 0.02%; *p* < 0.001). More specifically, the percentage of total apoptotic cells in 0.1, 1, 5, and 10 μM baicalein pretreatment groups was 13.55 ± 0.12, 11.35 ± 0.01, 10.18 ± 0.03, and 3.9 ± 0.01%, respectively. The current study thus shows that baicalein pretreatment significantly reduces β-GP-induced VSMCs apoptosis in a dose-dependent manner (*p* < 0.001).

### 2.4. Effect of Baicalein in the Downregulation of Runx2-BMP-2 Biomarkers in β-GP-Induced VSMCs Calcification

**Figure 4:**
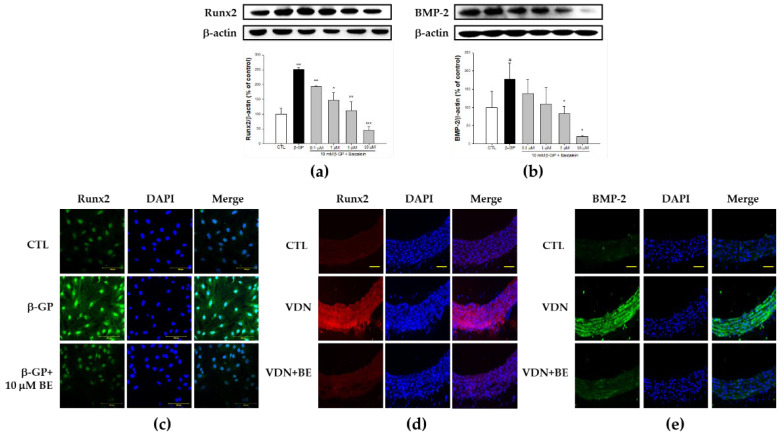
Baicalein suppresses the expression of osteochondrogenic-related proteins Runx2 and BMP-2 in rat VC. Western blots for (**a**) Runx2 protein and (**b**) BMP-2 protein. (**c**) The immunofluorescence detection of Runx2 protein using confocal microscope (200×) of primary rat VSMCs. The cells were pretreated with 10 μM baicalein for 1 h before addition of 10 mM β-GP-induced calcification for 48 h. Bar shows 100 μm. The immunofluorescence detection of (**d**) Runx2 and (**e**) BMP-2 proteins using confocal microscope (400×) in SD rat thoracic aorta tissue. Counterstaining of nuclei with DAPI in blue was merged into all images. Bar shows 25 μm. All values are presented as mean ± SEM. ^#^ *p* < 0.05; ^###^ *p* < 0.001 vs. control group (untreated VSMCs); * *p* < 0.05; ** *p* < 0.01; *** *p* < 0.001 vs. the β-GP group.

To define whether vascular calcification involves VSMCs transition to an osteoblast-like phenotype, we assessed the expression of Runx2, a master osteoblast transcription factor. Runx2 is a specific transcription factor that can induce osteoprogenitors or preosteoblasts to differentiate into osteoblasts and then proceed to regulate the maturation of osteoblasts [22,23]. Runx2 expression was significantly upregulated in the β-GP group compared with controls (*p* < 0.001). Conversely, as illustrated in Figure 4a, 0.1, 1, 5, and 10 μM baicalein pretreatment suppressed the expression of Runx2 in β-GP-induced VSMCs calcification in a dose-dependent manner (*p* < 0.001). To confirm the Runx2 expression of VSMCs culture, immunofluorescence staining of Runx2 was performed. As shown in Figure 4c, β-GP-induced VSMCs calcification causes increased expression of Runx2. Baicalein pretreatment results in decreased Runx2 expression. The nuclei were clearly stained with DAPI. As shown in Figure 4d, as with the in vitro study, immunofluorescence staining of Runx2 in the rat thoracic aorta demonstrated increased expression of Runx2 in VDN group. Baicalein pretreatment downregulated Runx2 expression in aortas of VC rats.

Next, we evaluated the expression of BMP-2 through western blotting assay. As shown in Figure 4b, in the calcification medium, the expression of BMP-2 was markedly higher than the control group (*p* < 0.05). Pretreatment with baicalein in certain doses significantly decreased the expression of BMP-2 (*p* < 0.05). Further, as presented in Figure 4e, BMP-2 expression was elevated in VDN-induced rat thoracic aorta calcification. Its expression was decreased by baicalein pretreatment. The present study indicates that baicalein pretreatment at various doses inhibits β-GP-induced VSMCs calcification through the integration signaling pathway of osteoblastic transcription factor Runx2 and BMP-2 in a dose-dependent manner both in vitro and in vivo.

### 2.5. Effect of Baicalein in the Elevation of SM22-α and α-SMA Expression in β-GP-Induced VSMCs Calcification

**Figure 5:**
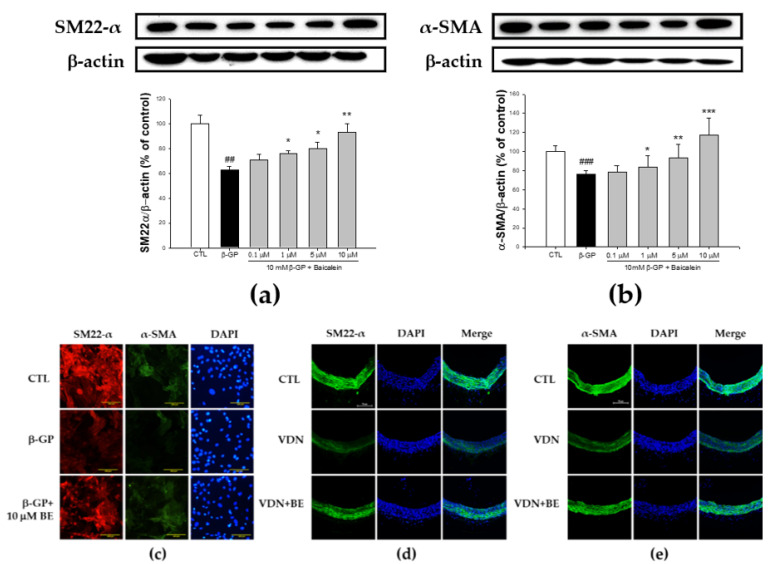
Baicalein enhances the expression of contractile-related proteins SM22-α and α-SMA in rat VC. Western blots for (**a**) SM22-α protein and (**b**) α-SMA protein. (**c**) The represent images of VSMCs were pretreated with 10 μM baicalein for 1 h before addition of 10 mM β-GP-induced calcification for 48 h. examined by double immunofluorescent staining. Bar shows 100 μm. The immunofluorescence detection of (**d**) SM22-α and (**e**) α-SMA proteins using confocal microscope (200x) in SD rat aorta tissue. Counterstaining of nuclei with DAPI in blue was merged into all images. Bar shows 50 μm. All values are presented as mean ± SEM. ^##^ *p* < 0.005; ^###^ *p* < 0.001 vs. control group (untreated VSMCs); * *p* < 0.05; ** *p* < 0.01; *** *p* < 0.001 vs. the β-GP group.

SM22-α and α-SMA are important markers of VSMCs contractile phenotype. To evaluate whether baicalein pretreatment increases vascular related contractility morphology in β-GP-induced VSMCs calcification, we measured the expression of SM22-α and α-SMA. In the calcification medium, compared with control group, both the percentage of SM22-α (62.44 ± 2.95%) and α-SMA (75 ± 4.99%) expressions were lower (as shown in Figure 5a,b). The percentage of SM22-α expression in 0.1, 1, 5, and 10 μM of baicalein pretreatment was 70.93 ± 4.15, 75.67 ± 2.54, 80.05 ± 4.72, and 92.87 ± 7.04%, respectively. Baicalein pretreatment significantly increased the SM22-α expression in β-GP-induced VSMC calcification (*p* < 0.01). The percentage of α-SMA expression in 0.1, 1, 5, and 10 μM of baicalein pretreatment was 78.82 ± 6.94, 83.76 ± 13.43, 93.14 ± 18.49, and 117.28 ± 23.25%, respectively. Baicalein pretreatment significantly improved α-SMA expression in VSMCs with β-GP-induced calcification (*p* < 0.01). As shown in Figure 5d, immunofluorescence staining of SM22-α in the rat thoracic aorta showed decreased expression of SM22-α in the VDN group. Baicalein pretreatment upregulated SM22-α expression in aortas of VC rats.

VSMCs are spindle-shaped with a prominent, centrally located nucleus. In the present study, we used immunofluorescence staining of SM22-α and α-SMA to define the VSMCs morphology. As illustrated in Figure 5c, high magnification showed filaments from the cell poles parallel to the long axis of the cells; there were considered as SM22-α-positive cells (red color). The nuclei were clearly stained by DAPI. For α-SMA, fluorescence microscopy showed filaments from the cell poles parallel to the long axis of the cells; there were considered as α-SMA positive cells (green color). The nuclei were clearly stained with DAPI. Further, as depicted in Figure 5e, α-SMA expression was reduced in rat thoracic aorta with VDN-induced calcification. Its expression was elevated by baicalein pretreatment. The present study indicates that baicalein pretreatment inhibits VSMCs calcification through the integration signaling pathway of osteoblastic transcription factor Runx2 and BMP-2 in a dose-dependent manner both in vitro and in vivo.

### 2.6. Effects of Baicalein on Cardiac Morphometry in VDN-Induced VC Rats

**Figure 6:**
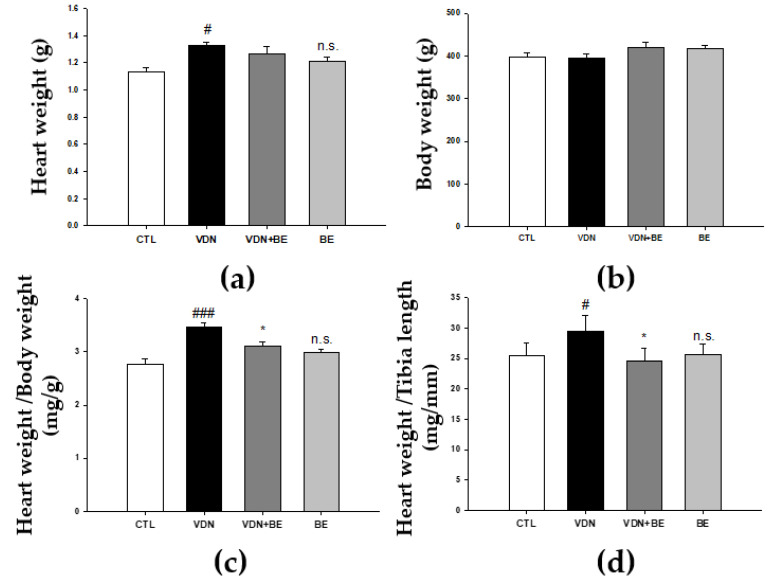
The effects of baicalein on heart and body weight in SD rats. (**a**) Heart weight (g), (**b**) body weight (g), (**c**) heart and body weight ratio (mg/g), and (**d**) heart weight and tibia length ratio (mg/mm). Each point represents the mean ± SEM, *n* = 10. ^#^ *p* < 0.05; ^###^ *p* < 0.001 compared with the control group; * *p* < 0.05 compared with VDN group; n.s. mean not significant vs. control group.

To determine whether baicalein has an effect on cardiac morphometry, we measured whole heart and body weight ratio. As shown in Figure 6a, VDN-induced VC rats’ heart weights were increased (*p* < 0.05) compared with the control group. No groups showed a difference in body weight (Figure 6b). We then evaluated the ratio of heart weight and body weight. As shown in Figure 6c, the VDN group had the highest heart to body weight ratio compared with other groups. The administration of 25 mg/kg BW baicalein significantly decreased the heart to body weight ratio in VDN + BE group (*p* < 0.05). Moreover, the ratio of heart weight and tibia length were higher compared with the control group (*p* < 0.05). Heart weight to tibia length ratio was lower in VDN + BE group (*p* < 0.05). This suggests that baicalein has an effect on cardiac morphometry in VDN-induced VC rats.

### 2.7. Effects of Baicalein on Left Ventricular Cardiac Performance in VDN-Induced Rats VC

**Figure 7:**
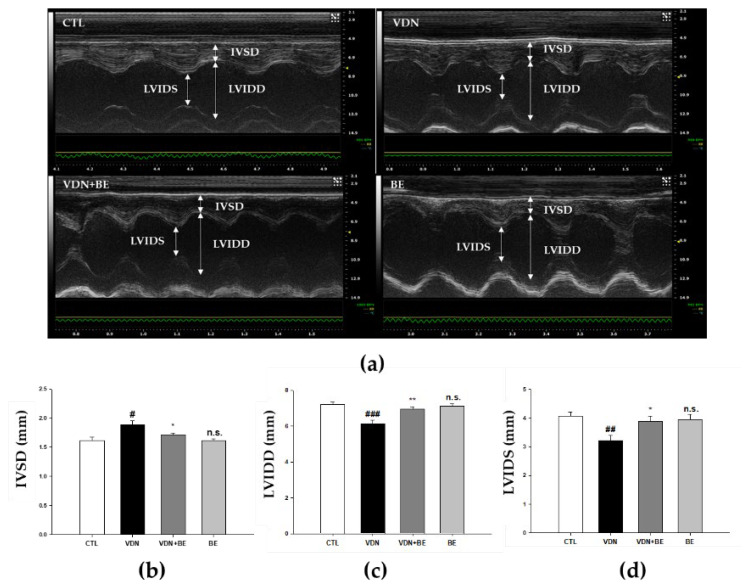
The effects of baicalein on cardiac performance in SD rats. The M-mode images were represented from individual groups by echocardiograph observation. (**a**) Echocardiogram, (**b**) IVSD, (**c**) LVIDd, and (**d**) LVIDs. Each point represents mean ± SEM, *n* = 10. ^#^ *p* < 0.01; ^##^ *p* < 0.01; ^###^ *p* < 0.001 vs. control group; * *p* < 0.05; ** *p* < 0.01 vs. VDN group; n.s. mean not significant vs. control group.

Figure 7a shows M-Mode echocardiogram representative images of SD rats. The IVSD was significantly increased in VDN-induced VC rats compared with the control (Figure 7b, *p* < 0.05). Baicalein pretreatment significantly reduced the IVSD compared with VDN group (*p* < 0.05). Both LVIDd and LVIDs were significantly decreased in VC rats with VDN-induced VC (Figure 7c,d, *p* < 0.01). Baicalein pretreatment resulted in an increase of these two parameters in VDN-induced VC rats. 

### 2.8. Effects of Baicalein in the Redox Status of VDN-Induced Rats VC

**Figure 8:**
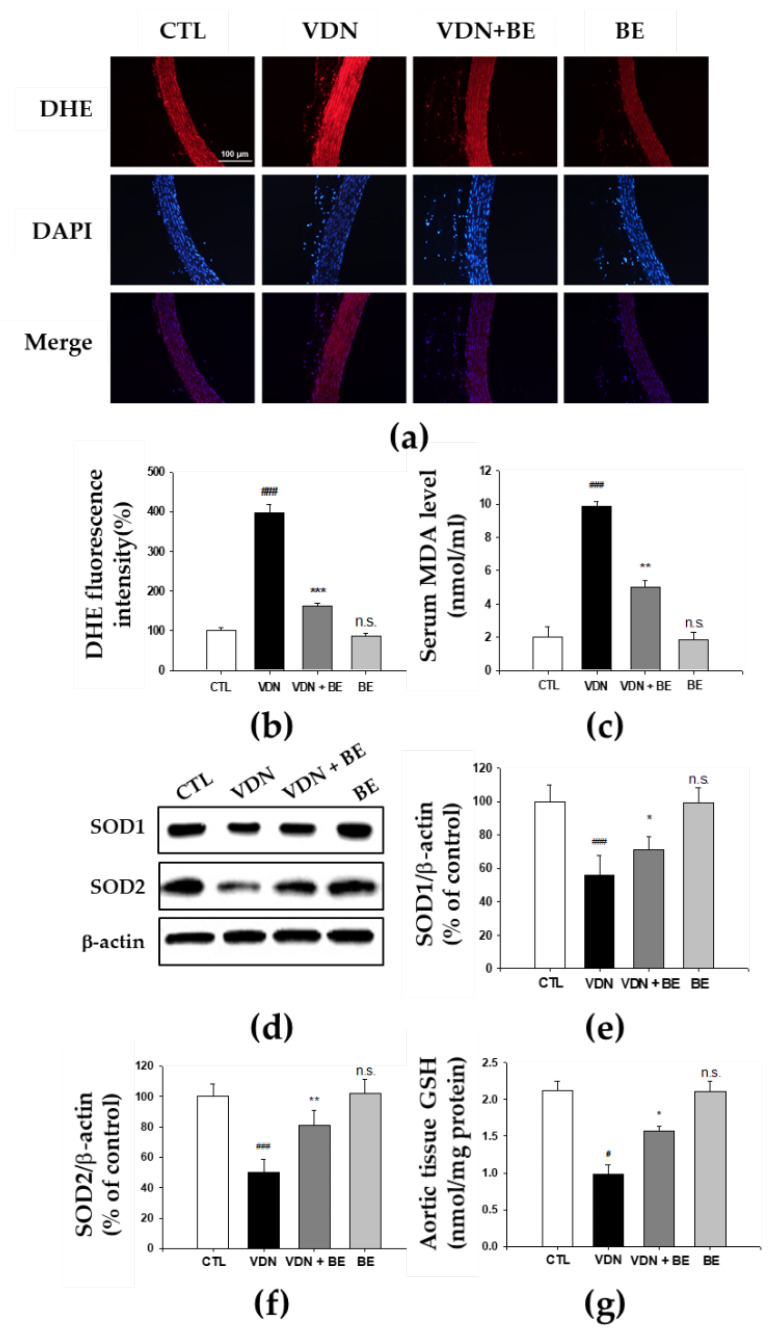
Effects of baicalein on VDN-induced oxidative stress markers in SD rats. (**a**) DHE staining to detect anion superoxide generation (200× magnification) through confocal microscopic observation of nuclear thoracic aorta wall. Counterstaining of nuclei with DAPI in blue was merged into all images. (**b**) Quantified DHE fluorescence intensity relative to number of nuclei. (**c**) Level of MDA in the serum. (**d**) The protein level of SOD1 and SOD2 in aortic homogenates. The quantitative analysis of (**e**) SOD1 and (**f**) SOD2 by western blotting. (**g**) Level of GSH in the aorta tissues. Each point represents the mean ± SEM, *n* = 10. ^#^ *p* < 0.05; ^###^ *p* < 0.001 compared with the control group. Bar shows 100 μm. ^###^ *p* < 0.001 compared with control group, * *p* < 0.05; ** *p* < 0.05; *** *p* < 0.001 compared with VDN group; n.s. mean not significant vs. control group.

We evaluated aortic superoxide generation as detected by DHE staining to investigate whether baicalein inhibits the in situ oxidative stress. The increased level of DHE fluorescence in the VDN group as shown in Figure 8a. Superoxide production in thoracic aorta was 100 ± 7.24 a.u (arbitrary units), 397.07 ± 21.64 a.u, 162.61 ± 6.77 a.u, and 86.53 ± 8.32 a.u, respectively in CTL, VDN, VDN + BE, and BE. The VDN group had the highest generation of superoxide, and it is significant when compared to the CTL group (*p* < 0.001). Baicalein pretreatment reduced superoxide production in rats with VDN-induced VC (*p* < 0.001). In addition, as shown in Figure 8b, MDA level of the thoracic aorta homogenates in the VDN group was 9.6 ± 0.25 nmol/mL. This was significantly higher compared with the control group (2.17 ± 0.19 nmol/mL, *p* < 0.001). Baicalein pretreatment led to the decrease of MDA level in the thoracic aorta tissues (5 ± 0.0 nmol/mL, *p* < 0.001). These findings suggest that baicalein inhibits oxidative stress in rats with VDN-induced VC. Further, to evaluate endogenous antioxidant activity, we measured aortic SOD1, SOD2, and glutathione (GSH). Our results show that baicalein pretreatment increased these protein expressions in rats with VDN-induced VC.

## 3. Discussion

In the present study, we investigated whether baicalein inhibits vascular calcification through in vitro and in vivo models. To induce calcification in vitro, VSMCs were given 10 mM β-GP. However, a rat VC model was generated by administration of vitamin D3 plus nicotine (VDN model). We also evaluated the underlying mechanisms of the effect of baicalein in oxidative stress and apoptosis of VSMCs. The major novel findings of this study are: (1) Pretreatment of baicalein remarkably lowered levels of calcium and ALP activity both in vitro and in vivo. (2) The expression of BMP-2 and Runx2 decreased in a dose-dependent manner both in vitro and in vivo. (3) The expression of vascular contractile-related proteins: SM22-α and α-SMA increased in a dose-dependent manner both in vitro and in vivo. (4) Blockade of oxidative stress partially stimulated the beneficial effects of baicalein on vascular calcification by inhibiting oxidative stress, increasing endogenous vascular antioxidants: SOD1, SOD2, and GSH in vivo. Cells apoptosis was suppressed in vitro. Further, we observed cardiac morphometry through measurement of heart to body weight ratio, heart weight to tibia length, and echocardiography evaluation. (5) Baicalein pretreatment reduced the heart to body weight ratio in VDN-induced VC rats. (6) The left ventricular internal dimension (LVID), both LVIDs and LVIDd), were enhanced by 25 mg/kg BW baicalein pretreatment. Interventricular septum thickness in diastole (IVSD) was reduced by baicalein pretreatment. Taken together, our findings suggest that baicalein inhibits oxidative stress and death of VSMCs, and is associated with attenuation vascular calcification in models of VC via the Runx2 and BMP-2 pathway. The expressions of vascular phenotypic contractile markers (SM22-α and α-SMA) were also upregulated. Left ventricular hypertrophy was reduced, and the left ventricular chamber was enhanced by baicalein pretreatment in a VC rat model. 

Vascular calcification is the pathological accumulation of hydroxyapetite in the vascular system and is associated with atherosclerosis, diabetes, certain heredity conditions, and kidney disease, especially chronic kidney disease (CKD) contributing to its high mortality [1]. The search for treatments that will prevent or reverse vascular calcification is ongoing. One approach is to look for agents which inhibit pro-calcification factors and processes which facilitate active mineralization of the vasculature [3]. Dietary phytochemicals are believed to afford some protection against vascular calcification. There is increasing interest in understanding the mechanisms of action of nutritional compounds with health benefits, known as nutraceuticals [24]. The classification of phytochemicals is broadly divided into certain classes that include the alkaloids, the terpenoids, and the phenylpropanoids. Flavonoids, which comprise the largest class of dietary nutraceuticals, are derived from the general phenylpropanoid pathway. Flavonoids are classified into subclasses that include the flavanones, the flavones, the flavonols, the isoflavones, the anthocyanins, and the proanthocyanidins or condensed tannins [25]. The rhizome of *Scutellaria baicalensis*, the source of baicalein used in our study, is rich in flavones. Previous studies of dietary intake of flavones among different populations varied from 0.7 to 9.0 mg/d in adults in Europe [26], 1.1 to 1.6 mg/d in women in the United States [27], and 1.9 to 4.2 mg/d in female adolescents in China [28]. Recent studies highlight that flavone have demonstrated many potentially advantageous properties through in vitro, in vivo and human trials [14].

A growing body of literature has examined the mechanisms of vascular calcification including loss of endogenous inhibitors, degradation of extracellular matrix, and induction of apoptosis and vesicle release followed by osteogenic differentiation of VSMCs [29,30,31]. Certain stimuli, such as increased levels of calcium or phosphate, VSMCs lead to the transformation of VSMC into an osteogenic phenotypic where the cells acquire features of chondrocytes and osteoblasts [31]. Osteogenic VSMCs exhibit an elevated expression of osteogenic markers, such as alkaline phosphatase, BMP-2, and Runx2, but show diminution of calcification-inhibitor protein expression [32]. Exposure to high levels of calcium results in intracellular calcium overload in VSMCs leading to microcalcification and ultimately macrocalcification, which contributes to vascular stiffness and remodeling [33]. 

The present study confirms the effectiveness of the flavone baicalein in protecting VSMCs against β-GP-induced calcification. Several other studies using in vitro and in vivo models have found that baicalein demonstrates efficacy in the inhibition of vascular calcification [34,35]. Flavones are one of the flavonoid subclasses which occur in aromatic herbs-parsley, celery, garlic, green peppers, chamomile tea, and some cereals such as millet and wheat [36]. In the current study, baicalein inhibited VC as reflected by a decrease in calcium present and a reduction of osteoblast-related differentiation protein BMP-2 and osteoblast-related transcriptional factor, Runx2. Several factors including hypercalcemia and hyperphosphatemia are thought to be promoters of vascular calcification [37]. In the regulation of vascular contraction, as well as cell structure and function, calcium is a critical signaling mediator of VSMCs [1]. It is therefore unsurprising that alteration of calcium homeostasis is associated with certain vascular disease states. For example, hypercalcemia, as found in CKD patients, can drive VSMC calcification. Disturbance of intracellular calcium homeostasis can lead to depletion of calcification inhibitors, increased calcium loading and deposition within microvesicles [38]. Growing evidence suggests that hyperphosphatemia and elevated phosphate ion product may also promote vascular calcification and are significantly linked to all-cause and cardiovascular disease mortality in patients with CKD [39]. Higher phosphate levels are associated with switching of VSMCs to an osteochondrogenic phenotype [1,40]. The presence of phosphate in vascular smooth muscle cells also induces an increase of ALP activity, and is linked to differentiating osteoblasts and hypertrophic chondrocytes. Increased ALP activity results in suppression of osteogenic inhibitors, such as dephosphorylating osteopontin and degrading pyrophosphate [41,42]. Further, elevated calcium and phosphate act synergistically to promote vascular calcification [41,43]. In this study, we found that baicalein reduced both the levels of calcium, and ALP activity, in vascular calcification in a dose-dependent manner. To the best of our knowledge, our study is the first investigation to find baicalein exerting a protective effect on VC. However, what is the molecular mechanism by which baicalein inhibits VC correlates at least partially with oxidative stress. An imbalance of ROS-generating systems and antioxidant systems produces oxidative stress. It leads to VC pathogenesis, such as phosphate imbalance, VSMC differentiation, inflammatory reaction, DNA damage, and extracellular matrix remodeling [42]. Oxidative stress stimulates phenotypic changes of VSMCs into osteochondrogenic-like cells mediated by upregulation expression of Runx2/Cbfa1 (core-binding factor alpha-1), osteopontin, RUNX2, BMP-2, and β-GP. The involvement of β-GP in the osteoblastic differentiation of VSMCs mainly through the activation of Cbfa-1 and NOXs. Baicalein reduces ROS-generating systems and stimulates antioxidant systems. It prevents phenotypic changes of VSMCs into osteochondrogenic-like cells mediated by the expression of RUNX2 and BMP-2.

In the present study, baicalein had some protective effects against apoptosis in β in VSMCs with β-GP induced calcification. Emerging evidence has shown that apoptosis of VSMC promotes vascular calcification [44,45]. The upregulation of apoptosis induced by β-GP was significantly reduced by baicalein at a dose-response manner. It is known that chronic apoptosis of vascular smooth muscle cells accelerates atherosclerosis and promotes calcification and medial degeneration [46], and it has been suggested that apoptosis is a key mechanism promoting vascular tissue mineralization [8]. Previous studies have shown that high extracellular calcium and phosphate levels induce apoptosis of VSMCs. Under such conditions, VSMCs undergo apoptosis and release apoptotic bodies, which then induce the formation of calcium phosphate deposition [38]. Calcium regulates key components of vesicles in the vascular smooth muscle cell-derived matrix vesicles to enhance mineralization. Apoptosis leads to increase VSMC mineralization and promotes vascular calcification. Inhibition of apoptosis results in reducing VSMC mineralization and restoring vascular calcification [8,44,46,47]. Our findings provide further evidence for the use of baicalein to inhibit β-GP induced VSMC calcification through down-stream reduction of apoptosis.

Concomitantly, VSMCs undergo a phenotypic alteration characterized by upregulation of genes for bone differentiation including osteocalcin, osteopontin, and Runx2, and the production of osteoblastic proteins such as BMP-2 and BMP-4 [47]. As a member of the TGF-β family, BMP-2, promotes osteoblast differentiation and maturation, participates in the development and reconstruction of bone and cartilage and accelerates the repair of bone defects [48,49]. Our findings validate the usefulness of baicalein in preventing β-GP induced VSMCs calcification through downregulation of Runx2 expression, a marker for VSMC transition to a calcifying phenotype. Furthermore, baicalein suppressed the production of BMP-2, an osteoblastic protein. 

To test whether baicalein enhances the vascular contractility in a VC model, we evaluated the production of vascular contractility-related proteins through in vitro and in vivo studies. Upon calcification-induced vascular injury, VSMC responds by losing contractility markers and differentiate to a synthetic phenotype. Certain protein such as SM22-α and α-SMA are pivotal in maintaining vascular contractility. Quantitative changes in these structural proteins are associated with the alteration of VSMC contractility phenotype, and hence the contraction and dilatation of the vasculature [50]. Our study shows that baicalein treatment increases the production of SM22-α and α-SMA in a VC model. It provides evidence of a preventive role of baicalein by upregulation of vascular contractility-related proteins and inhibition of VSMC remodeling.

The amelioration of oxidative stress appears to be central to how baicalein protects against VC in our model. Oxidative stress occurs when the generation of free radicals exceeds the capacity of the organism’s antioxidant enzymes to ‘disarm’ them. Reactive oxygen species or free radicals are produced by both normal cellular metabolism as well as in disease states, and react with biomolecules such as proteins, lipids, and DNA to cause cellular damage. Oxidative stress has been implicated in contributing to cell damage and death in many pathological conditions. It has been reported that oxidative stress plays a central role in aggravating organ damage, including that of the heart, kidneys, bones, and aortas of diabetic and CKD model rats [51,52,53,54]. Excessive oxidative stress has emerged as an important mediator promoting VC through mechanisms such as VSMC differentiation, inflammation, DNA damage, and extracellular matrix remodeling [55]. Oxidative stress is associated with calcium deposition in blood vessel walls. Elevated ROS production was found to be predominately around calcifying foci [56]. Oxidative stress has been demonstrated in hyperphosphatemia-induced VC. A previous study provided additional clues indicating that mitochondrial ROS-induced nuclear factor-κB (NF-κB) signaling activation played a central role in hyperphosphatemia-induced VC and high Pi induced VSMC calcification in rats with chronic renal failure [11]. Moreover, increases in oxidative stress upregulates Runx2 mRNA which results in BMP-2 activation. BMP-2 modulates bone formation and osteoblast differentiation [57]. Indeed, when produced in excess, O• reacts with nitric oxide (NO) to produce the toxic species ONOO− which promotes a variety of negative effects on cellular function. These include alteration of transcription factors, kinases, protein synthesis, and redox-sensitive genes, which in turn influence endothelial function, increase vascular contractility, vascular smooth muscle cell growth and apoptosis, monocyte migration, lipid peroxidation, inflammation, and increased deposition of ECM proteins, all major processes deeply involved in the pathogenesis and progression of vascular damage in cardiovascular disease [57]. Physiologically, ROS generation is tightly regulated by endogenous cellular antioxidants, which include SOD, catalase, thioredoxin, glutathione, and antioxidant vitamins. In physiological conditions, the rate of ROS generation is counterbalanced by the rate of elimination. In contrast, under pathological conditions, including hypertension, ROS are produced in concentrations that cannot be controlled by the usual protective antioxidant mechanisms, leading to a state of oxidative stress [58]. Several experimental studies have indicated that various antioxidant approaches can attenuate the progression of organ damage in cardiovascular conditions [59]. Antioxidants are able to protect human vascular smooth muscle cells from oxidative stress-related calcification. In this study, our findings demonstrated that baicalein reduces superoxide radicals and lipid peroxidation marker MDA, and stimulated endogenous antioxidants SOD1, SOD2, and GSH. They can also represent the oxygen concentration of surrounding tissues. Oxygen concentration is a potential modulator of mineralization tendency. Thus, previous studies would suggest that baicalein reduces VC by suppressing aortic wall oxidative stress and, as a result, preventing the contingent phenotypic changes and apoptosis of VSMC in the aortic media. 

**Figure 9:**
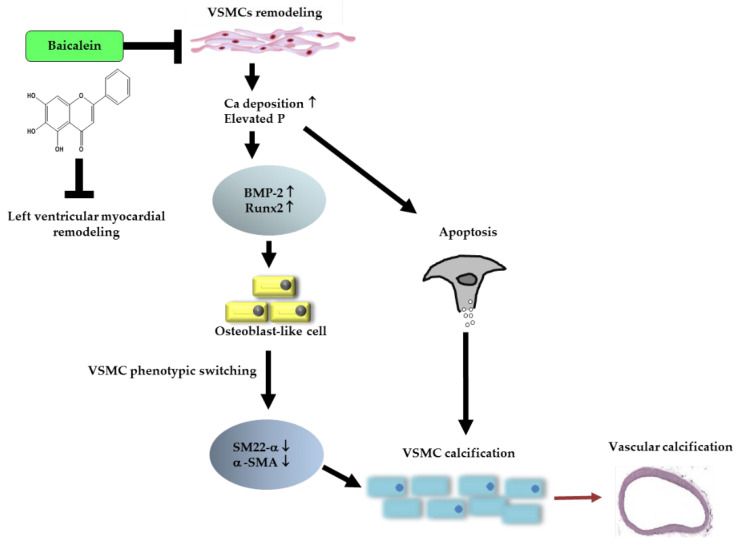
Proposed mechanisms of baicalein inhibits vascular calcification. Baicalein ameliorates calcium and phosphate homeostasis, leading to the downregulation of Runx2 and BMP-2 expressions. Furthermore, baicalein blocks VSMC calcification by restoring the SM22-α and α-SMA; VSMC-related contraction biomarkers. Somewhat surprisingly, we found that baicalein pretreatment inhibits cardiac remodeling.

Our findings provide important evidence about baicalein as an inhibitor of vascular calcification through its effects on oxidative stress, tissue and cell calcium deposition, VSMC apoptosis, expression of BMP-2 and Runx2, and expression of vascular contractile-related proteins SM22-α and α-SMA. Of concern, in our study baicalein appeared to prevent LV myocardial remodeling (Figure 9). Myocardial modification changes LV distensibility; the ability of LV to contract and dilate to maintain LV performance and supply enough blood and oxygen to the body. Baicalein pretreatment caused smaller heart weight, prevented LV hypertrophy and enhanced the function of LV to contract and dilate simultaneously on systole and diastole. Baicalein pretreatment particularly supported the heart to maintain cardiac output in a rat VC model. The results of the present study cannot be transferred to humans, nonetheless they provide further evidence that baicalein is a promising agent for the prevention of vascular calcification. Although our study sheds light on some of the pathways by which it may achieve this, more work is required to further elucidate the pathways and their molecular mechanisms.

## 4. Materials and Methods

### 4.1. Reagents

Baicalein was purchased from Sigma-Aldrich (product number 465119, Saint Louis, MO, USA). Its standard product is 270.24 g/mol, with a purity of no less than 98%. We dissolved the baicalein in phosphate buffered saline (PBS, Thermo Fisher Scientific Inc., Waltham, MA, USA) to make stock solutions and stored them at −20 °C. β-glycerophosphate (β-GP) was obtained from Sigma-Aldrich (Saint Louis, MO, USA. To establish a VC rat model, vitamin D3 and nicotine were given. Vitamin D3 and nicotine were obtained from Sigma-Aldrich (Saint Louis, MO, USA).

### 4.2. Preparation and Culture of Primary Rat VSMCs 

At the age of 6 weeks, 25 SD rats were housed in a standard animal housing facility, subjected to a 12 h dark-light cycle and received standard commercial food and drinking water ad libitum. Room temperature was between 18 and 22 °C with humidity between 45–70%. Rat VSMCs was obtained from thoracic aorta of male SD rats as previously described [60]. VSMCs were cultured in Dulbecco’s Modified Eagle Medium (DMEM, Thermo Fisher Scientific Inc., Waltham, MA, USA) containing 10% fetal bovine serum (FBS) and 1% penicillin/streptomycin (Sigma-Aldrich, Saint Louis, MO, USA) at 37 °C with 5% CO_2_. The purity of the VSMCs cultures was confirmed by immunocytochemical localization of α-SMA. After confluence, the cells were sub-cultured using trypsin. Culture media were changed every three days and VSMCs in passages 3 to 5 were used for the experiments.

### 4.3. In Vitro VSMCs Calcification

To induce in vitro VSMCs calcification, we cultured VSMCs with DMEM containing 10 mM β-GP, 100 μg/mL ascorbic acid, and 10% FBS (both Sigma-Aldrich, Saint Louis, MO, USA) after pretreatment with the vehicle or several concentrations of baicalein in the medium for 1 h. VSMCs were cultured for 10 days and then harvested for analysis.

### 4.4. Alkaline Phosphatase (ALP) Activity

To measure ALP activity, cells and aorta tissues were washed with PBS and lysed in 0.1 M NaHCO_3_-Na_2_CO_3_ buffer containing 0.1% Triton X-100, 2 mM MgSO_4_, and 6 mM 4-nitrophenyl phosphate for 1 h at room temperature. ALP activity was determined using a QuantiChrom^TM^ Alkaline Phosphatase Assay Kit (DALP-500, BioAssay Systems, Hayward, CA, USA). The reaction was stopped by adding 1 M NaOH. The absorbance was detected an ELISA reader with absorbance at 405 nm (Dynex Technologies, Denkendorf, Germany). We ensured equal amounts of protein in both the cell samples and the aortic tissue samples by means of the Pierce^TM^ BCA protein Assay kit (Thermo Scientific, Waltham, MA, USA).

### 4.5. Alizarin Red S Staining and Calcium Content Quantification

Alizarin red S staining (Sigma-Aldrich, Saint Louis, MO, USA) was performed to detect calcium deposition. After fixation with 75% ethanol, cells were stained with 2% Alizarin red S solution for 1 h at room temperature. The cells were incubated in 10% cetylpyridinium chloride solution (Sigma-Aldrich, Saint Louis, MO, USA) for 30 min. The absorbance of the released Alizarin red S was measured using enzyme-linked immunosorbent assay (ELISA) at 540 nm. Calcium content of cells and aorta tissue lysates were determined using QuantiChrom^TM^ Calcium Assay kit (DICA-500, BioAssay Systems, Hayward, CA, USA). The absorbance was detected using an ELISA reader at 612 nm (Dynex Technologies, Denkendorf, Germany). 

For the in vivo study, histological slides of aortic wall tissue were dried, rinsed rapidly in distilled water, and placed in an Alizarin red S staining solution for 10 min at room temperature. Any unbound stain was removed from the slides which were then photographed under an optical microscope (Nikon ECLIPSE TE2000-S).

### 4.6. Flow Cytometric Analysis 

Apoptotic and necrotic cells were quantified using the flow cytometric analysis and an Annexin V-conjugated Alexa Fluor 488 Apoptosis Detection Kit (Molecular Probes, Eugene, OR, USA) based on the manufacturer’s instructions. To detect the percentage of apoptotic cells, the samples were stained simultaneously with Alexa Fluor^®^ 488 dye and propidium iodide (PI). The percentage of apoptotic cells were quantified using flow cytometry (Coulter Epics XL-MCL; Beckman Coulter, Indianapolis, IN, USA). These cell populations can be distinguished using a flow cytometer with the 488 nm line of an argon-ion laser for excitation.

### 4.7. Western Blot Analysis

Cells and aorta tissue extracts (30 μg/mL of protein) were subjected to 10% SDS-PAGE gels and transferred to nitrocellulose by electroblotting. Primary antibodies against Runx2 (1:1000, sc-10758, Santa Cruz, Dallas, TX, USA), α-SMA (1:1000, #2547, Sigma-Aldrich, Saint Louis, MO, USA), SM22-α (1:1000, ab14106, Abcam, Cambridge, MA, USA), BMP-2 (1:1000, ARG57829, Arigo, Santa Cruz, Dallas, TX, USA), SOD1 (1;1000, GTX100554, GeneTex, Irvine, CA, USA), and SOD2 (1;1000, GTX116093, GeneTex, Irvine, CA, USA) were applied, followed by horseradish peroxidase (HRP)-linked secondary antibodies (Millipore, St. Louis, MO, USA) for 1 h at room temperature. Protein detection was visualized by chemiluminescence reagents (Millipore, St. Louis, MO, USA) and captured with a charged-couple device (CCD) camera (MiniChemi- Chemiluninescence, Sage Creation Sciences, BioRiver, Co., Ltd., Beijing, China). The immunoreactive bands were quantitatively determined using ImageJ software. Internal control was confirmed by β-actin (1:1000, sc-47778, Santa Cruz, Dallas, TX, USA). 

### 4.8. Immunofluorescence Assay

VSMCs grown in 6-well plates were pre-treated with 0.1, 1, 5, and 10 μM of baicalein for 1 h, respectively. Subsequently, the medium was changed into β-GP for 48 h. Cells were washed with PBS and then were fixed with 4% paraformaldehyde at room temperature for 30 min. This was followed by permeabilization with 0.1% Triton X-100 for 5 min at 4°C. Non-specific binding of fixed cells was blocked with PBS containing 10% Bovine Serum Albumin (BSA) for 1 h at room temperature, and followed by incubation with primary antibodies overnight at 4 °C. After the cells were washed, bright fluorescent conjugated secondary antibodies was incubated in the cells for 1 h at room temperature. DAPI (4′,6-diamidino-2-phenylindole) was used to stain the cell nuclei. The fluorescence picture was visualized using a confocal laser scanning microscope coupled with an image analysis system (Olympus Fluoview FV1000, Olympus Optical Co., Tokyo, Japan).

Rat thoracic aorta tissues were separated, immersed in saccharose (30% *w/v*), embedded in optimal cutting temperature (OCT), and stored at −20 °C until immunofluorescence analyze was performed. Ten micron thick sections were cut in OCT blocks with a cryostat and mounted on polylysine-coated glass slides. Subsequently, the slides were incubated with 10% paraformaldehyde solution. After washout in PBS plus 0.05% Triton X-100, the sections were blocked for 30 min with 5% BSA and then incubated with rabbit antibodies against BMP-2, Runx2, α-SMA, and SM22-α overnight at 4 °C. The sections were then incubated with a secondary Goat Anti-Rabbit IgG Antibody, (H+L) FITC conjugate (dilution 1:100; Sigma-Aldrich, Saint Louis, MO, USA), and nuclei were stained with 4, 6-diamidino-2-phenylindole (DAPI, Sigma-Aldrich, Saint Louis, MO, USA) at room temperature. Sections were mounted and viewed with a confocal laser-scanning microscope (Olympus Fluoview FV1000, Tokyo, Japan).

### 4.9. Experimental Animals

SD rats were purchased from BioLASCO Taiwan Co., Ltd., Taipei, Taiwan. The experiments were conducted in accordance with the ARRIVE (Animal Research: Reporting of in vivo experiments) guidelines. The VC technique in SD rats was based on previous methods [60,61,62]. Six-week-old male SD rats (180–200 g) were randomly divided into four groups: control (CTL, *n* = 10), model (VDN, *n* = 10), baicalein treatment (VDN + BE, *n* = 10), and baicalein only (BE, *n* = 10). Rats in the control group received both oral gavage and intramuscular injection of corn oil. The VDN and VDN + BE rats were administrated 25 mg/kg body weight of nicotine in 5 mL corn oil orally (Sigma-Aldrich, Saint Louis, MO, USA), plus intramuscular injection of vitamin D3 (3 × 105 IU/kg BW, Sigma-Aldrich, Saint Louis, MO, USA) at 9 a.m. on day 1. Nicotine administration was repeated after 8 h. Baicalein was given orally at 25 mg/kg BW once every two days for 4 weeks, both in VDN + BE and BE groups. The dose and duration of baicalein treatment was selected based on the previous published literature with minor modifications showing significant protection against cisplatin-induced acute kidney injury in mice when orally administered baicalein at a dose of 25 mg/kg body weight for 14 consecutive days [63]. During the treatment period, body weight of each animal was recorded weekly; the heart index (heart weight to body weight ratio) was measured at sacrificed. At the end of study, all animals were sacrificed by intraperitoneal 30% urethane injection.

All rats were allowed free access to food and water during the acclimatization and experimental period. The rats were kept in a well-ventilated room at standard experimental conditions with room temperature of 22 degrees (±2 °C), relative humidity of 60% (±10%) and a 12 h light/dark cycle. 

### 4.10. Echocardiography 

At the end of study, all rats underwent cardiac morphology evaluation using a Vevo 2100TM high-resolution in vivo imaging system. As per a prior study, the echocardiography scans were all performed by the same sonographer and each measurement was calculated from five consecutive cardiac cycles [64]. Before echocardiography, all rats were anesthetized with Isoflurane (Panion & BF Biotech Inc., Taipei, Taiwan). They were secured to a board, shaved to facilitate sonography, and an ultrasound transonic gel EcoGel 100TM (Eco-Med Pharmaceutical Inc. Mississauga, ON, Canada) was applied to the thoracic skin. A 5–12 MHz echocardiography probe was used to obtain an M-mode trace of the left ventricle (LV), enabling measurement of LVIDd, LVIDs, and IVSD.

### 4.11. Determination of In Vivo Redox Status

To analyze redox status, we measured aortic superoxide generation, aortic glutation, and conducted enzymatic analysis of plasma MDA. Dihydroethidium staining (Thermo Fisher Scientific Inc., Waltham, MA, USA) was used to measure in situ levels of superoxide (O^2−^) in the rat thoracic aorta as previously described [64]. DHE is freely permeable to cells, and in the presence of superoxide is oxidized to fluorescent ethidium bromide which is trapped intracellularly by intercalation into the DNA. The thoracic aorta frozen sections (10 μm) on polylisine glass slides were incubated with 10 μM DHE at 37 °C for 30 min in a humidified chamber protected from light. Fluorescent images of ethidium bromide were obtained using confocal laser-scanning microscope (Olympus Fluoview FV1000, Tokyo, Japan) and the number of nuclei labelled by DHE was automatically counted in each field with image-analysis software (Zeiss-Axio vision software). 

To assay plasma MDA (ab118970, Abcam, Cambridge, MA, USA), blood samples were collected from left ventricular blood at sacrificed. Blood was centrifuged at 3000 rpm for 10 min at 4°C and then measured according to the manufacturer’s protocols. To assay aortic glutathione (GSH, K464-100, BioVision, Natick, MA, USA), one frozen segment of thoracic aorta tissue homogenate was cut into small pieces in ice-cold RIPA (radioimmunoprecipitation assay) lysis buffer (M-PER^®^, Thermo Fisher Scientific Inc., Waltham, MA, USA) mixed with 1% Triton-X100 and then underwent sonication. Supernatant was collected from the homogenates, after which it was centrifuged at 13,000× *g* for 30 min at 4 °C. The GSH content were measured from the total cellular protein obtained from the supernatant. Data were measured using a V-5100H spectrophotometer (BioTek Instruments, Inc., Winooski, VT, USA). The GSH absorbance was at 450 nm wavelength. 

### 4.12. Statistical Analysis

Results were expressed as mean ± standard error of measurement (SEM). To determine the significance of differences between two groups, Student’s *t*-test was used. For multiple comparisons, we used a one-way ANOVA test followed by Tukey post-hoc test via SigmaPlot 10 (Systat Software, Inc., San Jose, CA, USA). Data was considered significant at *p* < 0.05.

## 5. Conclusions

Our study demonstrates that baicalein, at least in part, potentially inhibits vascular calcification in a rat model by preventing apoptosis, suppressing the Runx2-BMP-2 signaling pathways, and preserving a vascular contractility phenotype via stimulation of SM22-α and α-SMA production. The study provides further evidence of a preventive role for baicalein in vascular calcification which could, if reproducible in human subjects, contribute to the development of treatment strategies to reduce the burden of cardiovascular disease.

## Data Availability

Not applicable.
